# Mental Health Screening in General Practices as a Means for Enhancing Uptake of Digital Mental Health Interventions: Observational Cohort Study

**DOI:** 10.2196/28369

**Published:** 2021-09-16

**Authors:** Alexis E Whitton, Rebecca Hardy, Kate Cope, Chilin Gieng, Leanne Gow, Andrew MacKinnon, Nyree Gale, Kathleen O'Moore, Josephine Anderson, Judith Proudfoot, Nicole Cockayne, Bridianne O'Dea, Helen Christensen, Jill Maree Newby

**Affiliations:** 1 Black Dog Institute Randwick Australia; 2 University of New South Wales Randwick Australia

**Keywords:** depression, anxiety, general practice, screening, digital mental health

## Abstract

**Background:**

Digital mental health interventions stand to play a critical role in managing the mental health impact of the COVID-19 pandemic. Thus, enhancing their uptake is a key priority. General practitioners (GPs) are well positioned to facilitate access to digital interventions, but tools that assist GPs in identifying suitable patients are lacking.

**Objective:**

This study aims to evaluate the suitability of a web-based mental health screening and treatment recommendation tool (*StepCare*) for improving the identification of anxiety and depression in general practice and, subsequently, uptake of digital mental health interventions.

**Methods:**

StepCare screens patients for symptoms of depression (9-item Patient Health Questionnaire) and anxiety (7-item Generalized Anxiety Disorder scale) in the GP waiting room. It provides GPs with stepped treatment recommendations that include digital mental health interventions for patients with mild to moderate symptoms. Patients (N=5138) from 85 general practices across Australia were invited to participate in screening.

**Results:**

Screening identified depressive or anxious symptoms in 43.09% (1428/3314) of patients (one-quarter were previously unidentified or untreated). The majority (300/335, 89.6%) of previously unidentified or untreated patients had mild to moderate symptoms and were candidates for digital mental health interventions. Although less than half were prescribed a digital intervention by their GP, when a digital intervention was prescribed, more than two-thirds of patients reported using it.

**Conclusions:**

Implementing web-based mental health screening in general practices can provide important opportunities for GPs to improve the identification of symptoms of mental illness and increase patient access to digital mental health interventions. Although GPs prescribed digital interventions less frequently than in-person psychotherapy or medication, the promising rates of uptake by GP-referred patients suggest that GPs can play a critical role in championing digital interventions and maximizing the associated benefits.

## Introduction

### Background

Anxiety and depression are leading causes of disability worldwide, and in the wake of the COVID-19 pandemic, the personal, social, and economic costs of these conditions are expected to rise [1]. Although effective interventions exist, these conditions often go untreated [2]. This poses a significant problem for individuals and health care systems, as anxiety and depression are associated with reduced quality of life [3], increased suicide risk [4], and increased health service use [5]. Although increasing access to evidence-based mental health care seems to be an obvious solution, achieving this in the context of an already overstretched mental health system represents a major challenge.

Digital mental health interventions (eg, web-based cognitive behavioral therapy), used alone or in blended models of care, have been highlighted as crucial tools for addressing unmet mental health treatment needs [1]. Indeed, health policies in the United Kingdom [6] and Australia [7] recommend digital mental health interventions as the first-line treatment for individuals with mild or subsyndromal depression. These interventions have been shown to be as effective as in-person psychotherapy for a range of disorders [8] and can be delivered with minimal or no therapist input [9]. Furthermore, these interventions can promote improvements in symptoms that are sustained over several years [10], provide immediate support (ie, no waiting time), and are free from many barriers that prevent individuals from accessing face-to-face treatment (eg, cost, inconvenience, and stigmatization). However, the integration of digital mental health interventions into routine mental health care has been slow [11]. New strategies are needed to promote a greater uptake of digital mental health tools.

One way to increase the integration of digital mental health interventions into routine mental health care is to better support general practitioners (GPs) in prescribing these interventions to their patients. Digital mental health interventions can provide significant benefits in the general practice setting because GPs encounter high rates of common mental health conditions, with 1 in 4 patients experiencing depression [12] and 1 in 5 experiencing anxiety [13]. Furthermore, GPs are often an individual’s first point of contact with mental health services and are a trusted source of information for patients, so they are well placed to facilitate greater access to, and confidence in, digital tools for mental health. However, two major challenges have prevented the adoption of digital mental health tools in general practice. First, although mental health conditions are common among general practice attendees, only half of all patients with a mental health condition are recognized by their GP as having one [14]. Accordingly, low rates of disorder detection may prevent GPs from identifying patients who are suitable candidates for digital mental health interventions. Second, given the broad array of digital apps and tools marketed for mental health, GPs report a lack of confidence in prescribing digital interventions and uncertainty about their evidence base inhibits their routine use [15].

To address these challenges, the Black Dog Institute developed StepCare, a web-based tool for use in general practice that offers digital mental health screening and treatment recommendations [16,17]. StepCare aims to facilitate the delivery of a stepped care approach to mental health care in the general practice setting. It does so by screening patients in the GP waiting area using a tablet device and then stratifying patients into groups according to symptom severity. StepCare then provides a screening report and set of treatment recommendations to GPs, which are matched in intensity to a patient’s specific level of need. Importantly, evidence-based digital mental health interventions are incorporated into the treatment recommendations for patients who screen positive for mild or moderate depressive or anxious symptoms, and GPs are supported in referring their patients to these interventions via referral links embedded in the program.

Initial studies of feasibility [16] and implementation [17] demonstrated that StepCare was acceptable to GPs and feasible for delivery in general practice settings. Moreover, two recent randomized controlled trials (RCTs) of similar digital patient stratification tools further support the utility of using digital solutions to deliver stepped care in the general practice setting. Specifically, an RCT evaluating an eHealth platform (*Target-D*) that aimed to support patients in managing depression found that digitally stratifying patients and providing matched interventions resulted in greater improvements in depressive symptoms over 3 months compared with usual care [18]. Similarly, an RCT of a similar digital decision support tool (*Link-me*) demonstrated that digitally facilitated stepped care resulted in greater improvements in psychological distress in general practice patients compared with usual care [19].

### Objectives

A key goal of the StepCare tool was to increase the use of digital mental health interventions in general practice by (1) helping GPs to identify patients who would be suitable candidates for digital interventions (ie, via screening for patients with mild to moderate symptoms), (2) assisting GPs in determining when a digital mental health intervention should be used alone or in combination with a higher-intensity intervention (eg, a psychologist), and (3) reducing GPs’ uncertainty surrounding which digital interventions to prescribe and how to introduce them to the patient in the limited time available during a consultation. StepCare has been implemented in 85 general practices across Australia, and more than 5000 patients were screened from July 2017 to March 2020. The aim of this study is to examine whether StepCare is a suitable means for identifying patients who may be candidates for digital mental health interventions and to promote the uptake of these interventions in the general practice patient population.

## Methods

### Sample and Recruitment

Information about the StepCare tool was disseminated to primary health networks (PHNs) via conferences, workshops, and networks. A total of 8 PHNs from New South Wales, Australian Capital Territory, and Victoria signed up to use StepCare during the study period (July 24, 2017, and March 31, 2020), and each PHN invited expressions of interest from general practices in their region. In total, 85 general practices expressed interest, and all GPs within these practices were invited to participate. The GPs who provided informed consent to participate were then provided with information and training from either their PHN or staff from the Black Dog Institute on how to implement StepCare in their practice. Both PHNs and the Black Dog Institute staff provided ongoing support to practices throughout the implementation period.

Adult patients who were attending a GP appointment at one of the participating practices, regardless of the reason for their visit, were invited to participate in the screening. When patients presented to the reception staff, they were handed a mobile tablet that displayed an information and consent page, and patients who agreed to participate in screening indicated their consent via button click on the tablet. To assess their eligibility for screening, an initial set of questions was administered that confirmed that the patient was aged ≥18 years, had not undergone screening in the past 6 months, and could provide either a mobile phone number or email address (required for patients to receive follow-up monitoring assessments to track symptom change over time).

### Ethics Statement

This study was approved by the University of New South Wales Human Research Ethics Committee (HC15827).

### Design

This study used an uncontrolled, observational, prospective cohort design.

### Screening Measures

Depressive symptoms were screened for using the 9-item Patient Health Questionnaire (PHQ-9) [20]. Total scores range from 0 to 27. StepCare defines severity levels as nil-minimal (0-4), mild (5-9), moderate (10-19), and severe (20-27). Item 9 was used to assess thoughts about suicide and self-harm. The presence of suicidal thoughts was flagged to the GP in a separate column on the patients’ screening report, where a score of 1 on item 9 of the PHQ-9 indicated thoughts of mild severity, a score of 2 as moderate, and a score of 3 as severe. Anxiety symptoms were screened for using the 7-item Generalized Anxiety Disorder (GAD-7) scale [21]. Total scores range from 0 to 21, with severity levels defined as nil-minimal (0-4), mild (5-9), moderate (10-14), and severe (15-21).

StepCare also included 2 items that assessed whether the patient had previously discussed mental health issues with their GP and whether their current appointment was about mental health. This served as a proxy indicator of whether a patient was likely to be known to their GP as having a mental health condition. Patients were also asked sociodemographic questions, including whether they were Aboriginal or Torres Strait Islander; a carer for children, someone with a disability, someone with a chronic illness, or someone who was frail-aged; spoke a language other than English; had concerns about their accommodation or housing; and what their alcohol use habits were (Alcohol Use Disorders Identification Test) [22]. The PHQ-9 and GAD-7 scores informed the StepCare treatment recommendations; however, all responses were transmitted to GPs for consideration during the consultation.

### Screening Procedure

On presentation to the practice, the reception staff provided adult patients with an internet-enabled mobile tablet featuring the StepCare information and consent page. Patients could either consent or decline to participate by clicking a button. Those who consented were asked to provide their contact details and complete the sociodemographic questions, the PHQ-9, the GAD-7, and the Alcohol Use Disorders Identification Test. The patient’s screening results were sent directly to their GPs’ medical inbox using a secure messaging service for review during the consultation.

### Treatment Recommendations

StepCare treatment recommendations were stratified according to the patient’s symptom severity. Digital mental health interventions were incorporated into Steps 1 and 2 as follows.

#### Step 1: Web-Based Self-Help

The self-guided digital mental health intervention myCompass [23] was recommended for patients with mild symptoms. MyCompass has been shown to be effective in improving symptoms of depression and anxiety over 7 weeks relative to waitlist and attention control conditions [23] and has more than 50,000 registered users to date.

#### Step 2: Guided Web-Based Therapy or Face-to-Face Psychotherapy (Consider Medication)

Guided web-based therapy via the MindSpot Clinic or face-to-face therapy with a clinical psychologist was recommended for patients with moderate symptoms. MindSpot offers therapist-guided web-based courses and has been found to produce significant improvements in depression and anxiety [24,25]. The Step 2 recommendation also suggested that the GP consider pharmacotherapy.

#### Step 3: Face-to-Face Psychotherapy, Antidepressant Medication (Consider a Psychiatrist)

Referral to a clinical psychologist and pharmacotherapy were recommended for patients with severe symptoms. The GP was encouraged to consider referral to a psychiatrist for pharmacotherapy management.

#### Managing Suicidality

For patients who scored 1 or higher on item 9 of the PHQ-9, StepCare also provided GPs with several options to assist them in supporting patients who reported suicidal thoughts, including providing prompts to help initiate a discussion with the patient regarding their responses on the screener, steps for further assessing risk, information describing how to develop a safety plan for patients who are actively suicidal, and links to relevant local and national crisis services.

### GP Prescribing Patterns

During the consultation, GPs were asked to record the treatment they prescribed by filling in checkboxes accessible via the patient’s screening report. Although GPs were strongly encouraged to record prescribed treatments, to minimize the burden on GP workflow, this was not mandatory.

### Patient Outcomes and Treatment Use

Individuals who screened positive for depression or anxiety were invited to complete follow-up assessments every 2 weeks for 18 weeks to monitor symptoms (those with nil-minimal symptoms were not followed up). These follow-up assessments captured key outcome measures, including depressive symptom severity (PHQ-9) and anxiety symptom severity (GAD-7). In addition to these outcome measures, patients were asked to indicate, via checkboxes, which of a series of mental health treatments they had used in the past 2 weeks. Follow-up assessments were not mandatory, and patients could opt out of receiving assessment reminders at any time.

Each time a patient completed a follow-up assessment, a report was sent to their GP’s medical inbox. This report displayed the patient’s PHQ-9 and GAD-7 scores, their score on item 9 of the PHQ-9 (indicating suicidal thoughts), and a line graph showing symptom improvement from baseline. Accompanying this report was also a series of alerts that notified GPs of patients who showed signs of improvement, as well as those who showed evidence of deterioration, severe symptoms that did not improve, and nonadherence to monitoring assessments. Improvement was defined as a patient whose PHQ-9 or GAD-7 scores had improved by at least one severity category compared with the previous fortnight; deterioration was flagged when a patient’s PHQ-9 or GAD-7 score increased by at least one severity category relative to the previous fortnightly period; severe and unchanging symptoms were flagged where a patient with PHQ-9 or GAD-7 scores in the severe range at baseline had not improved by at least one severity category by week 4; and nonadherence to assessments was flagged when a patient who screened positive for depressive or anxious symptoms at baseline had not completed two consecutive follow-up assessments. For patients who did not improve, or who deteriorated, the GP was encouraged to consider scheduling a follow-up appointment with the patient to review their treatment plan.

For the purposes of our statistical analyses, we defined *remitters* as individuals who were nil-minimal (0-4) on the PHQ-9 and GAD-7 at their last follow-up assessment, and *responders* as individuals who showed a decrease of at least one severity category on the PHQ-9 or GAD-7 at their last assessment.

### Statistical Analyses

Descriptive statistics were used to quantify patient symptoms, treatment use, and GP prescription patterns. The number needed to screen (NNS) was calculated using methods aligned with those adopted in multiarm clinical trials, as described by Rembold [26]. NNS is a statistic that is derived from the number needed to treat (NNT) statistic that is commonly reported in clinical trials assessing the effectiveness of an intervention and reflects the number of people who need to be screened to prevent one adverse event. However, it differs from NNT in that it also incorporates information about the prevalence of undetected diseases that can be potentially identified via screening. In our study, it was calculated by first computing the absolute risk (AR) of identifying undiagnosed symptoms of anxiety or depression through screening and the AR of identifying undiagnosed symptoms of anxiety or depression under a hypothetical *no screening* condition. For the purposes of our calculation, we assumed that in the hypothetical *no screening* condition, no individuals with undiagnosed symptoms of depression or anxiety would have been identified that would not have already been identified through care as usual without screening. Next, we computed the difference in AR under these two conditions (ie, the AR deduction [ARD]). Finally, we computed the NNS in the same manner as that used to compute NNT, which is the inverse of the ARD (ie, 1/ARD) [26].

This was supplemented with an intention-to-treat analysis examining symptom improvement over the follow-up period using data from all assessment points (mixed model for repeated measures analysis with random intercepts and slopes; implemented in Stata [version 13.1; StataCorp LP]). We also performed a completer analysis to examine the rates of response and remission.

### Role of the Funding Source

The funder had no role in the study design, data collection, analysis, manuscript writing, or in the decision to publish the manuscript.

## Results

### Sample Characteristics

Of the 5138 patients who were offered the tool, 3777 (73.51%) completed screening and 3314 (64.50%) met the eligibility criteria (sample characteristics are shown in Table 1).

**Table 1:** Characteristics of baseline sample (N=3314).

Characteristic	Participants
Age (years), mean (SD)	43.4 (17.0)
Female, n (%)	2316 (69.89)
Aboriginal or Torres Strait Islander, n (%)	165 (4.98)
English as second language, n (%)	423 (12.76)
Cares for children, n (%)	1079 (32.56)
Cares for frail or disabled individuals, n (%)	305 (9.20)
Accommodation issues, n (%)	94 (2.84)
Seeing general practitioner for mental health reasons, n (%)	745 (22.48)
Seen general practitioner for mental health reasons previously, n (%)	1519 (45.84)

### Suitability of StepCare as a Screening Tool

#### Prevalence and Severity of Symptoms Identified

In total, 1428 individuals with symptoms of anxiety or depression were identified through screening (detection rate: 1428/3314, 43.09%). Of the baseline sample, 13.82% (458/3314) had mild symptoms, 17.68% (586/3314) had moderate symptoms, and 11.59% (384/3314) had severe symptoms. Furthermore, just under one third (454/1428, 31.79%) of symptomatic individuals reported suicidal ideation or thoughts of self-harm. Overall, depressive symptoms (1333/3314, 40.22%) were more common than anxiety symptoms (855/3314, 25.80%). There were no differences in the proportion of males and females who screened positive for either depressive or anxious symptoms (both values of *P*>.18).

For patients who screened in the mild symptom severity range, most screened positive for depressive symptoms only (346/458, 75.5%) with fewer screening positive for anxiety symptoms only (54/458, 11.8%) or both depressive and anxious symptoms (49/458, 10.7%). In contrast, as symptom severity increased, patients were more likely to screen positive for both depressive and anxious symptoms. Specifically, in the moderate range, 59.6% (349/586) screened positive for both depressive and anxious symptoms [depressive symptoms only (202/586, 34.5%); anxious symptoms only (28/586, 4.8%)], and in the severe range, 94.3% (362/384) screened positive for both depressive and anxious symptoms [depressive symptoms only (7/384, 1.8%); anxious symptoms only (13/384, 3.4%)].

#### Previously Unidentified or Untreated Mental Health Symptoms

Of the 1428 patients who screened positive for depressive or anxious symptoms, 335 (23.46% of symptomatic sample or 10.11% of patients overall) had never seen their GP for mental health reasons, including at the time of screening. These previously unidentified or untreated patients were older than symptomatic patients who had previously seen their GP for mental health reasons (44.73 vs 38.58; *t*_1,426_=6.36; *P*<.001). Most unidentified or untreated patients had mild (184/335, 54.9%) or moderate symptoms (116/335, 34.6%); however, 1 in 10 had severe symptoms (35/335, 10.4%) or reported suicidal ideation or thoughts of self-harm (49/335, 14.6%), indicating that StepCare also identified patients with significant unmet mental health treatment needs.

#### Results for NNS

Of the 5138 patients who underwent screening, 335 (6.52%) had anxious or depressive symptoms that were previously unidentified or untreated. Accordingly, the AR for recognizing previously unidentified symptoms via screening was AR_screening_=335/5138=0.0652. Although this was not an RCT, under a hypothetical *no screening* condition, we can assume that none of these individuals would have been detected, yielding an AR of:

AR_no screening_=0/5138=0

Therefore, the ARD can be calculated as AR_screening_–AR_no screening_:

ARD=335/5138–0/5138=0.0652

An NNS can be calculated as the inverse of the ARD:

NNS=1/0.0652=15.3 (95% CI 13.9-17.1)

This indicates that for every 16 patients who are offered mental health screening, 1 individual with previously unidentified or untreated depressive or anxious symptoms will be identified.

### Ability of StepCare to Facilitate Uptake of Digital Mental Health Interventions

#### GP Prescribing Patterns

GP prescription data were available for 23.39% (334/1428) of patients. These included 30.2% (101/334) patients with mild symptoms, 41.9% (140/334) with moderate symptoms, and 27.8% (93/334) with severe symptoms. Separate independent samples 2-tailed *t* tests showed that baseline PHQ-9 and GAD-7 scores did not differ between patients whose GP did and did not provide prescribing data (both values of *P*>.54).

Although digital and high-intensity (ie, psychologists, psychiatrists, and pharmacotherapy) interventions were recommended by the StepCare tool at roughly equal rates (digital interventions were recommended for 241/334, 72.2% of patients; high-intensity interventions were recommended for 233/334, 69.8% of patients; categories not mutually exclusive), GPs prescribed high-intensity interventions nearly twice as often as low-intensity digital interventions. Specifically, a high-intensity intervention was prescribed by the GP for 56.3% (188/334) of patients, whereas a digital mental health intervention was prescribed by the GP for 30.8% (103/334) of patients. A side-by-side comparison of the treatments recommended by StepCare, relative to the treatments prescribed by GPs, is shown in Table 2. Furthermore, GP prescription patterns among patients with different levels of symptom severity are shown in Table 3.

**Table 2:** Comparison of StepCare treatment recommendations for all symptomatic patients (n=1428) versus GP^a^ prescribed treatments in symptomatic patients with GP prescribing data available (n=334).

Treatment^b^	Proportion of all symptomatic patients (n=1428) who were recommended a specific treatment by StepCare, n (%)	Proportion of symptomatic patients with GP prescribing data (n=334) who were prescribed a specific treatment by their GP, n (%)
Web-based self-help	458 (32.1)	60 (18)
Guided web-based therapy	586 (41)	54 (16.2)
Psychologist	970 (67.9)	135 (40.4)
Medication	970 (67.9)	109 (32.6)
Psychiatrist	384 (26.9)	31 (9.3)

^a^GP: general practitioner.

^b^Column percentage totals do not sum to 100% as patients could be prescribed multiple treatments or no treatment at all.

**Table 3:** General practitioner prescribing patterns as a function of patient baseline symptom severity (N=334).

Treatment prescribed by general practitioner^a^	Patients with mild symptoms (n=101), n (%)	Patients with moderate symptoms (n=140), n (%)	Patients with severe symptoms (n=93), n (%)
Prescribed web-based self-help	33 (32.7)	15 (10.7)	12 (12.9)
Prescribed guided web-based therapy	11 (10.9)	28 (20)	15 (16.1)
Prescribed psychologist	22 (21.8)	59 (42.1)	54 (58.1)
Prescribed medication	8 (7.9)	50 (35.7)	51 (54.8)
Prescribed psychiatrist	2 (2)	8 (5.7)	21 (22.6)

^a^Percentages reflect the percentage of patients in each symptom severity category; column percentage totals do not sum to 100% as patients in the same symptom severity category could be prescribed multiple treatments or no treatment at all.

#### Patient Treatment Use Patterns

Treatment use data were available for 42.44% (606/1428) of patients. Most patients (525/606, 86.6%) reported the use of at least one form of mental health treatment during the follow-up period. Patients with treatment use data had higher baseline PHQ-9 (t_1426_=3.98; *P*<.001; mean 12.81, SD 6.16) and GAD-7 scores (t_1408_=3.77; *P*<.001; mean 9.43, SD 6.59) than patients who did not provide treatment use data (PHQ-9 mean 11.50, SD 6.18; GAD-7 mean 8.10, SD 6.59), indicating that the following patterns may be more applicable to patients with more severe symptoms.

**Figure 1:**
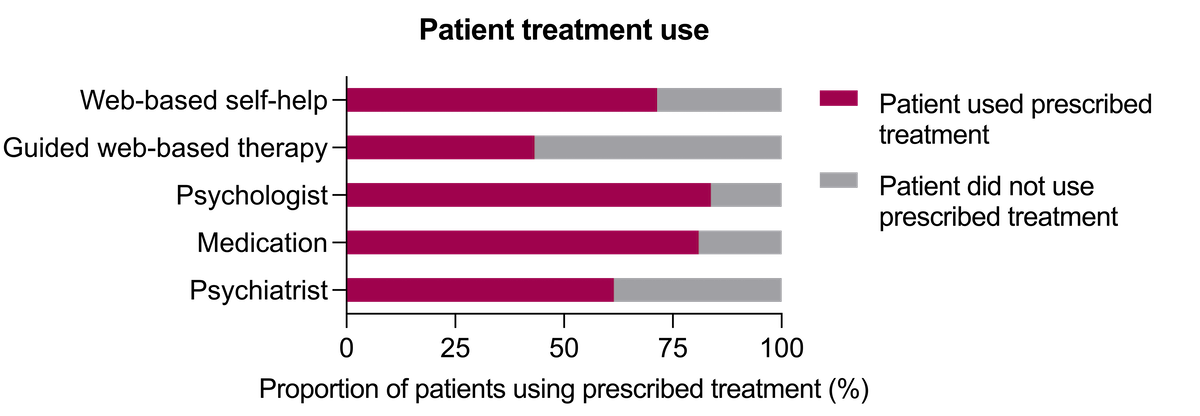
Data show the rate at which patients used the treatment that was prescribed to them by their general practitioner. Values are expressed as a proportion of symptomatic patients for whom both patient adherence and general practitioner prescribing data were available (n=165).

Of the 606 patients who provided treatment use data, 165 (27.2%) also had GP prescribing data available, which allowed us to determine whether a patient used the treatment prescribed by their GP. More than two-thirds of patients (35/52, 67%) who were prescribed a digital mental health intervention (either alone or in combination with a high-intensity intervention) reported using one of these digital interventions over the follow-up period. The rates of treatment uptake were also high for high-intensity interventions; nearly all patients (96/102, 94.1%) who were prescribed a high-intensity intervention by their GP reported that they had used a high-intensity intervention over the follow-up period. The patient use of GP-prescribed interventions is shown in detail in Figure 1.

### Patient Outcomes

#### Symptom Improvement Over Time

The intention to treat (mixed model for repeated measures) analysis revealed a significant main effect of *time* for the PHQ-9 (*P*<.001), where scores decreased by an average of 3.62 points from baseline to week 18 (moderate to mild severity range; Cohen *d*=0.58 computed using baseline SD of 6.20). Comparisons between baseline and follow-up PHQ-9 scores (Table 4) showed that symptom severity at each follow-up point was significantly lower than the score at baseline (all *P*s<.001). Similar effects were observed for the GAD-7, where the main effect of *time* was significant (*P*<.001), with scores decreasing by an average of 1.45 points from baseline to week 18 (upper end of mild to lower end of mild severity range; Cohen *d*=0.22, baseline SD 6.62). Comparisons between baseline and follow-up GAD-7 scores (Table 5) showed that symptom severity at each follow-up point except weeks 2 and 4 was significantly lower than at baseline (all values of *P*<.001).

**Table 4:** Changes in depressive symptom severity (9-item Patient Health Questionnaire) over time.

Time point	Estimated marginal, mean (SE)	Modeled change from baseline, mean (95% CI^a^)	*P* value^b^
Baseline	12.05 (0.16)	N/A^c^	N/A
Week 2	10.22 (0.22)	­−1.84 (−2.23 to −1.44)	<.001
Week 4	9.75 (0.24)	−2.31 (−2.75 to −1.86)	<.001
Week 6	9.15 (0.25)	−2.90 (−3.37 to −2.43)	<.001
Week 8	8.86 (0.26)	−3.19 (−3.70 to −2.69)	<.001
Week 10	8.81 (0.28)	−3.24 (−3.78 to −2.69)	<.001
Week 12	8.76 (0.29)	−3.30 (−3.87 to −2.73)	<.001
Week 14	8.33 (0.31)	−3.72 (−4.31 to −3.13)	<.001
Week 16	8.42 (0.32)	−3.63 (−4.25 to −3.01)	<.001
Week 18	8.43 (0.33)	−3.62 (−4.27 to −2.97)	<.001

^a^The 95% CI refers to the change from baseline.

^b^*P* values indicate the significance level for comparisons between baseline scores and scores at each of the follow-up assessments.

^c^N/A: not applicable.

**Table 5:** Change in anxious symptom severity (7-item Generalized Anxiety Disorder scale) over time.

Time point	Estimated marginal, mean (SE)	Modeled change from baseline, mean (95% CI^a^)	*P* value^b^
Baseline	8.67 (0.18)	N/A^c^	N/A
Week 2	8.56 (0.20)	−0.11 (−0.51 to 0.29)	.58
Week 4	8.26 (0.21)	−0.41 (−0.83 to 0.01)	.06
Week 6	7.86 (0.22)	−0.81 (−1.27 to −0.36)	<.001
Week 8	7.63 (0.24)	−1.04 (−1.54 to −0.55)	<.001
Week 10	7.33 (0.25)	−1.34 (−1.85 to −0.83)	<.001
Week 12	7.27 (0.25)	−1.40 (−1.92 to −0.88)	<.001
Week 14	7.18 (0.27)	−1.49 (−2.05 to −0.93)	<.001
Week 16	7.48 (0.30)	−1.20 (−1.81 to −0.58)	<.001
Week 18	7.22 (0.30)	−1.45 (−2.07 to −0.83)	<.001

^a^The 95% CI refers to the change from baseline.

^b^*P* values indicate the significance level for comparisons between baseline scores and scores at each of the follow-up assessments.

^c^N/A: not applicable.

#### Rates of Remission and Response

Completer analyses focused on symptomatic patients who completed at least one follow-up assessment (708/1428, 49.58%). Of this sample, 26.1% (185/708) scored in the asymptomatic range on the PHQ-9 and GAD-7 at follow-up and were classified as *remitters*. Furthermore, 55.4% (392/708) of cases dropped down at least one severity category on the PHQ-9 or GAD-7 and were considered *responders*. In contrast, 26.1% (185/708) increased by one or more severity categories during the follow-up period.

## Discussion

### Principal Findings

This study examined the performance of a digital mental health screening and treatment recommendation tool (*StepCare*) for improving the identification of depression and anxiety in general practice and for promoting the uptake of digital mental health interventions. Four key findings emerged. First, screening revealed a high prevalence of common mental health conditions among general practice patients, with 40.2% screening positive for depression and 25.8% screening positive for anxiety (cf. Australian population 12-month prevalence estimates of 6.2% for depression and 14.4% for anxiety) [27]. Nearly 1 in 4 of these patients had never consulted their GP about their mental health previously, indicating that screening identified individuals who may have otherwise been undetected. Second, the majority (89.5%) of untreated patients were in the mild to moderate range, demonstrating that screening, when followed by appropriate assessment, could assist GPs in identifying suitable candidates for digital mental health interventions. Third, although digital mental health interventions and high-intensity interventions were recommended by the StepCare tool at approximately equal rates, GPs favored high-intensity interventions over digital interventions. Fourth, although digital interventions were prescribed less often, more than two-thirds of patients who were prescribed a digital intervention by their GP reported using it. This confirms that GPs are well placed to facilitate greater uptake of digital mental health interventions.

Our findings align with prior studies showing high rates of depressive and anxious symptoms in general practice populations and the ability for mental health screening to identify patients with untreated symptoms [14]. These results extend prior research in two significant ways: (1) by estimating the NNS to detect previously untreated patients with depressive or anxious symptoms in an Australian general practice setting (NNS=16) and (2) by showing that a significant proportion of untreated patients have symptoms in the mild to moderate severity range, where digital mental health interventions may be especially useful. Although the impact of mental health screening on patient outcomes can only be determined via RCTs, our findings suggest that screening could identify important opportunities to implement low or no cost digital interventions, thereby reducing the burden on higher-intensity services.

Our finding that 1 in every 16 patients invited for screening had unidentified or untreated depressive or anxious symptoms positions mental health screening favorably when compared with other diseases routinely screened for in primary care. For example, type 2 diabetes screening for adults aged ≥40 years yields a detection rate of 1 in 32 [28], breast cancer screening for women aged 50 to 69 years yields a detection rate of approximately 1 in 94 [29], and cervical cancer screening in women aged >20 years yields a detection rate of approximately 1 in 143 [30]. Given that suicide outranks cancer as the leading cause of death for Australians aged 25 to 44 years [31] (the mean age of our sample was 43 years), mental health screening of the general practice population could significantly reduce suicide-related mortality.

We note that the rates of depressive and anxious symptoms in our study were higher than the population prevalence estimates of depression and anxiety, and there are several possible reasons for this. First, population prevalence estimates are based on individuals who meet the diagnostic criteria for depression or anxiety rather than individuals who demonstrate a positive result on a brief screening measure of depressive or anxious symptoms. In our study, it is likely that a subset of patients who screened positive for depression or anxiety would not have met the diagnostic criteria for depression or anxiety in further follow-up assessments with their GP. Second, it is likely that some participants who screened positive were experiencing transient symptoms rather than a persistent condition, with prior studies indicating that approximately one-fifth of patients who screened positive for depression in primary care no longer met the criteria after 2 weeks [32]. Third, the PHQ-9 and GAD-7 cutoff values we used were designed to capture milder subsyndromal symptoms in addition to more severe symptomatology; hence, the symptomatic sample in our study likely encompasses patients with milder symptoms compared with individuals who are included in epidemiological studies.

A fourth factor that likely underpinned our higher prevalence rates was the fact that several practices used StepCare to selectively screen high-risk patients (as recommended by the Royal Australian College of General Practitioners) [33] as opposed to implementing universal screening. In addition to biasing screening toward more symptomatic patients, this observation raises an important question regarding the best implementation of screening programs in general practice. Although selectively screening high-risk patients (eg, postpartum women) may increase the cost-effectiveness of screening, this approach may have the unintended effect of exacerbating existing disparities in access to mental health care [34]. For example, the Royal Australian College of General Practitioners guidelines indicate that patients at increased risk for depression are those who have a family history of psychiatric illness, have chronic medical conditions, are unemployed or of low socioeconomic status, have experienced significant life events, family violence or child abuse, or are part of the lesbian, gay, bisexual, transgender, and intersex community [33]. Although these factors may be known to the GP when there is a well-established relationship with the patient, this is less likely when a patient does not have a regular GP (up to 20% of patients) [35] or visits their GP infrequently (eg, indigenous patients or patients from rural areas) [36]. Accordingly, although risk-based screening may have lower opportunity costs than universal screening, it may fail to identify patients who may warrant further assessment by their GP and who may benefit from digital mental health interventions.

Regarding GP prescribing patterns, our findings suggest that although digital mental health interventions may be well suited to a substantial portion of the symptomatic general practice population, GPs show a preference for prescribing high-intensity treatments. Why might this be? Common barriers to use reported by GPs include uncertainty about a digital intervention’s evidence base and insufficient knowledge of how to refer a patient to a digital intervention [15]. However, StepCare was designed to overcome these barriers, as it only recommends empirically supported digital interventions and includes a referral pipeline that supports GPs in connecting their patients with the intervention. Additional barriers clearly exist and must be addressed. One by-product of the COVID-19 pandemic has been the rapid integration of telehealth services into general practices. This may help to break down attitudinal barriers to digital mental health tools; however, other practical changes, such as allowing GPs to formally itemize and charge for the time taken to discuss digital interventions with their patients, may also be needed to enhance their use in general practice.

### Limitations and Future Directions

This study has some limitations. First, this was an uncontrolled observational study of a tool used in a clinical setting. RCTs are needed to determine whether the opportunity costs of mental health screening in general practices are offset by superior patient outcomes. Second, GP prescribing data and patient treatment use data were available for only a portion of patients because completion of these surveys was optional. Greater tracking of prescription and treatment use patterns via electronic health records will be critical for providing a comprehensive perspective on digital intervention uptake. Finally, an important question that must be addressed in future research is whether patients who are prescribed digital mental health interventions show sufficient levels of engagement with these interventions to yield therapeutic benefits. If the rates of initial uptake are high when referred by a GP, but ongoing engagement remains low, then this may warrant consideration of alternative modes of delivery. Delivery methods that combine digital mental health interventions with face-to-face services, such as blended care [37-39], may be especially useful in this regard. Blended care has been found to promote greater adherence to digital programs, and preliminary evidence has demonstrated its efficacy in the general practice setting (eg, when guidance is provided by a nurse practitioner [40]).

### Conclusion

In conclusion, our findings indicate that a digital mental health screening and treatment recommendation tool may increase the opportunities to use digital mental health interventions in general practice. Leveraging these opportunities will be critical in addressing increased mental health treatment needs arising from the COVID-19 pandemic and in reducing existing disparities in access to affordable, evidence-based mental health care.

## Abbreviations

AR: absolute risk
ARD: absolute risk detection
GAD-7: 7-item Generalized Anxiety Disorder scale
GP: general practitioner
NNS: number needed to screen
NNT: number needed to treat
PHN: primary health network
PHQ-9: 9-item Patient Health Questionnaire
RCT: randomized controlled trial
